# Safrana l Prevents Prostate Cancer Recurrence by Blocking the Re-activation of Quiescent Cancer Cells *via* Downregulation of S-Phase Kinase-Associated Protein 2

**DOI:** 10.3389/fcell.2020.598620

**Published:** 2020-12-16

**Authors:** Xue Jiang, Yang Li, Ji-ling Feng, Wan Najbah Nik Nabil, Rong Wu, Yue Lu, Hua Liu, Zhi-chao Xi, Hong-xi Xu

**Affiliations:** ^1^School of Pharmacy, Shanghai University of Traditional Chinese Medicine, Shanghai, China; ^2^Shuguang Hospital Affiliated to Shanghai University of Traditional Chinese Medicine, Shanghai, China; ^3^Pharmaceutical Services Program, Ministry of Health, Petaling Jaya, Malaysia; ^4^Hospital Management Office, Shanghai University of Traditional Chinese Medicine, Shanghai, China

**Keywords:** safranal, prostate cancer, cell cycle re-entry, quiescent cancer cells, cancer recurrence, NF-κB, E2F1, Skp2

## Abstract

The re-proliferation of quiescent cancer cells is considered to be the primary contributor to prostate cancer (Pca) recurrence and progression. In this study, we investigated the inhibitory effect of safranal, a monoterpene aldehyde isolated from *Crocus sativus* (saffron), on the re-proliferation of quiescent Pca cells *in vitro* and *in vivo*. The results showed that safranal efficiently blocked the re-activation of quiescent Pca cells by downregulating the G_0_/G_1_ cell cycle regulatory proteins CDK2, CDK4, CDK6, and phospho-Rb at Ser807/811 and elevating the levels of cyclin-dependent kinase inhibitors, p21 and p27. Further investigation on the underlying mechanisms revealed that safranal suppressed the mRNA and protein expression levels of Skp2, possibly through the deregulation of the transcriptional activity of two major transcriptional factors, E2F1 and NF-κB subunits. Moreover, safranal inhibited AKT phosphorylation at Ser473 and deregulated both canonical and non-canonical NF-κB signaling pathways. Safranal suppressed the tumor growth of quiescent Pca cell xenografts *in vivo*. Furthermore, safranal-treated tumor tissues exhibited a reduction in Skp2, E2F1, NF-κB p65, p-IκBα (Ser32), c-MYC, p-Rb (Ser807), CDK4, CDK6, and CDK2 and an elevation of p27 and p21 protein levels. Therefore, our findings demonstrate that safranal suppresses cell cycle re-entry of quiescent Pca cells *in vitro* and *in vivo* plausibly by repressing the transcriptional activity of two major transcriptional activators of Skp2, namely, E2F1 and NF-κB, through the downregulation of AKT phosphorylation and NF-κB signaling pathways, respectively.

## Introduction

Tumor heterogeneity stems from the complex process of growth and diversity of cells such as quiescent cancer cells (QCCs), a subpopulation that transiently retreats from the cell cycle and arrests in the G_0_ phase (Yeh and Ramaswamy, 2015). QCCs have been clinically garnering attention as they can re-enter the cell cycle, resulting in cancer progression, recurrence, metastasis, and treatment resistance (Krall et al., 2018; Luskin et al., 2018; Recasens and Munoz, 2019). Prostate cancer (Pca) recurrence following primary treatment remains a leading clinical challenge, occurring in up to 50% of patients after 10 years of post-radical prostatectomy or radiotherapy (Hull et al., 2002). Hence, prevention of QCC re-awakening is an emerging paradigm for the treatment of Pca recurrence.

Consistent with the proposed treatment strategy for Pca recurrence, current research focuses on the mechanism of inhibiting the shift from a quiescent to a proliferative state. Mounting evidence indicates that inhibiting Rb-E2F1 signaling deregulates G_0_/G_1_-related cyclins, which eventually block QCCs from re-entering the cell cycle (Zetterberg et al., 1995; Yao et al., 2015; Xi et al., 2016; So and Cheung, 2018; Pennycook and Barr, 2020). c-MYC is a crucial regulator in cell cycle re-entry through the histone chaperone Facilitates Chromatin Transcription (Bi et al., 2019, 2020) and its E3 ligase FBXW7 (Xi et al., 2016). Additionally, Skp2 inhibition maintains quiescence (Zhang et al., 2019) and suppresses tumor progression in multiple transgenic mouse models (Agarwal et al., 2008; Lin et al., 2010; Wang et al., 2010). Nevertheless, a few effective leading compounds have impeded the transition from a quiescent to a proliferative state (Xi et al., 2016; Bi et al., 2020), emphasizing the necessity to mine for potential therapeutic options, which could block QCCs’ re-entry and hinder cancer recurrence.

Saffron, an age-old spice from the plant *Crocus sativus* L., is therapeutically valued in traditional Chinese and Ayurvedic medicine systems (Bhandari, 2015). Increasing evidence has established that safranal, a monoterpene aldehyde isolated from *C. sativus* (Tarantilis et al., 1994), exerts anticancer activities on various human malignancies (Samarghandian and Shabestari, 2013; Geromichalos et al., 2014; Samarghandian et al., 2014; Jabini et al., 2017; Al-Hrout et al., 2018; Cheriyamundath et al., 2018). However, the underlying antitumor mechanism of safranal relating to QCCs and cancer recurrence has not been established.

Therefore, the present study aimed to investigate the underlying mechanism of safranal and suppress the re-proliferation of quiescent Pca cells *in vitro* and *in vivo*, providing convincing evidence for the development of safranal as an agent against prostate cancer recurrence.

## Materials and Methods

### Cell Lines and Synchronization at Quiescence

Human Pca cell lines LNCaP and PC-3 were acquired from the American Type Culture Collection (Manassas, VA, United States) and cultured as previously described (Xi et al., 2016). Once the LNCaP cells attained a confluence of 70–80%, they were synchronized by changing to serum-free medium and cultured for 7 days to obtain a quiescent LNCaP model. In contrast, once the PC-3 cells reached 100% confluency, the medium was changed and retained for 3 days to achieve contact inhibition. Replenishing the serum for LNCaP cells and passaging PC-3 cells at lower confluency allowed the synchronized cells to re-enter the cell cycle. This model was used to simulate the progressive re-proliferation of Pca cells *in vivo*.

### Chemicals and Reagents

Safranal (≥90.0% purity) was purchased from Sigma Aldrich (St. Louis, MO, United States), dissolved in dimethyl sulfoxide (DMSO, Life Technologies, Carlsbad, CA, United States), and stored at -80°C. Propidium iodide (PI, P4170; Sigma-Aldrich), PrimeScript RT Reagent Kit (RR037A; TAKARA Biotechnology, Shiga, Japan), SYBR Green reagent (S-7563; Life Technologies), and SYBR Green Realtime PCR Master Mix (QPK-201; TOBOYO, Life Science, Osaka, Japan) were used. The antibodies for immunoblotting included those against CDK4 (#3830-1), CDK6 (#3524-1), and p21 (#3733-1) (Epitomics, Cambridge, United Kingdom). We acquired antibodies against p27 (sc528), IKKα/β (sc7607), α-tubulin (sc5286), and Skp2 (sc7164) from Santa Cruz Biotechnology (Dallas, CA, United States), while anti-β-actin antibody (#66009-1-Ig) was purchased from Proteintech (Wuhan, Hu Bei Province, China). The antibodies against p-Rb (Ser807/811, #9308), E2F1 (#3742), phospho-AKT (Ser473, #4060), AKT (#9272), phospho-IKKα/β (Ser176/180, #2697), phospho-IκBα (Ser32, #2859), IκBα (#4812), NF-κB p65 (#8242), and NF-κB2 (p100/52, #3017) were purchased from Cell Signaling Technology (Danvers, MA, United States), while those against lamin A/C (ab108595), CDK2 (ab32147), c-MYC (ab32072), and GAPDH (ab128915) were supplied by Abcam (Cambridge, United Kingdom).

### SYBR Green Assay

Quiescent LNCaP (1 × 10^4^ cells/well) and PC-3 (7 × 10^3^ cells/well) were induced to re-enter the cell cycle by seeding in 96-well plates containing a complete medium with the indicated concentrations of safranal. Additionally, the same number of cells was maintained at -80°C as baseline. After 72 h, the medium was discarded, 100 μl of lysis buffer [comprised of 20% radioimmunoprecipitation assay (RIPA) lysis buffer and 0.01% SYBR Green reagent] was added, and the mixture was incubated for 30 min in the dark. The frozen baseline cells were thawed and subjected to the above-mentioned lysis and incubation treatment as well as quantification of fluorescent SYBR Green-stained DNA, and calculation of growth inhibition (GI) concentrations at 50% (GI_50_) and 90% (GI_90_), with 95% confidence interval (CI), in LNCaP and PC-3 cells (Table 1) was performed as previously described (Xi et al., 2016).

**TABLE 1:** Growth inhibition (GI) concentrations of safranal with 95% confidence interval (CI) in LNCaP and PC-3 cells 72 h after release from quiescence.

GI%	LNCaP (mM)	95% CI	PC-3 (mM)	95% CI
GI_50_	0.133 ± 0.034	0.049–0.217	0.109 ± 0.002	0.102–0.115
GI_90_	0.239 ± 0.025	0.177–0.301	0.317 ± 0.045	0.205–0.428

*Quiescent LNCaP and PC-3 cells were re-activated and treated with various concentrations of safranal, and the rendered GI_50_ and GI_90_ with 95% confidence interval were determined using the SYBR Green assay. The GI values represent the mean ± SD of at least three independent experiments.*

### Flow Cytometric Analysis

Quiescent LNCaP and PC-3 cells were seeded into a six-well plate and treated with either safranal or DMSO for 24 h (PC-3) and 32 h (LNCaP). The cells were harvested and fixed overnight with ice-cold 70% ethanol in phosphate-buffered saline (PBS) at 4°C. Flow cytometric analysis with PI staining was performed as previously described (Li et al., 2018).

### Immunoblotting

Protein lysates were prepared by subjecting the cells to an ice-cold RIPA lysis buffer supplemented with protease/phosphatase inhibitor cocktail (#5872, Cell Signaling Technology, Danvers, United States). We conducted protein quantification, electrophoresis, and immunoblotting according to earlier protocols (Wong et al., 2019).

### Clonogenic Assay

Quiescent LNCaP (800 cells/well) or PC-3 (1 × 10^3^ cells/well) cells were seeded into six-well plates and treated with either DMSO or safranal (GI_50_ or GI_90_) for 24 and 48 h. Thereafter, the media were changed every 4–5 days. After 2 weeks, the colonies were fixed with 4% paraformaldehyde/PBS and stained with 1% crystal violet (Xi et al., 2016).

### Real-Time Reverse Transcription PCR

RNA was isolated with Trizol reagent, followed by reverse transcription with PrimeScript RT Reagent Kit and quantification using mRNA-specific primers in a StepOnePlus Real-Time PCR System (ABI) employing the SYBR Green Realtime PCR Master Mix. The details of the protocols were as previously described (Xi et al., 2016), and all results were normalized to TATA box-binding protein (TBP). The sequences of primers used were as follows: c-MYC forward, 5′-GCTGCCAAGAGGGTCA-3′ and reverse, 5′-CGCACAAGAGTTCCGTAG-3′; Skp2 forward, 5′-GAAACGGCTGAAGAGCAAAG-3′ and reverse, 5′-GGAG GCACAGACAGGAAAAG-3′; p21 forward, 5′- CTGGAGACT CTCTGCAGGGTCGAAA-3′ and reverse, 5′-GATTAGGGCTT CCTCTTGGAGAA-3′; p27 forward, 5′-GGCCTCAGAAGACG TCAAAC-3′ and reverse, 5′- ACAGGATGTCCATTCCATGA-3′; E2F1 forward, 5′-AGTTCATCAGCCTTTCCC-3′ and reverse, 5′-AGGTCCCCAAAGTCACAG-33′; NF-κB p65 forward, 5′-CC CACGAGCTTGTAGGAAAGG-3′ and reverse, 5′-GGATTCCC AGGTTCTGGAAAC-3′; NF-κB2 forward, 5′- TGAGAAGGAC ACCCGAAGC-3′ and reverse, 5′-GAGCAGCATTTAGCAGC AAG-3′; and TBP forward, 5′- GAACCACGGCACTGATTTTC-3′ and reverse, 5′-CCCCACCATGTTCTGAATCT-3′.

### Preparation of Nuclear and Cytoplasmic Extracts

We used a nuclear and cytoplasmic protein extraction kit (Beyotime Biotechnology, Shanghai, China) as per the manufacturer’s instructions. The isolated proteins were subjected to western blot analysis.

### Luciferase Reporter Assay

We sourced the pGM-E2F-Luc reporter plasmid from Genomeditech Biotechnology (Shanghai, China). pML-NFκB-Fluc2-Neo enhanced reporter plasmid (MA0504), renilla luciferase reporter plasmid pML-SV40-hRluc (MA0503), and dual luciferase reporter assay kit were purchased from Meilun Biology Technology Co. Ltd. (Dalian, China). The schematic diagram of pGM-E2F-Luc, pML-NFκB-Fluc2-Neo enhanced, and pML-SV40-hRluc reporter constructs is provided in Supplementary Figure 1. Following a 6-day serum withdrawal for LNCaP and a 2-day contact inhibition for PC-3 cells, the cells were transiently transfected with pGM-E2F-Luc, pML-NFκB-Fluc2-Neo enhanced, and renilla luciferase reporter plasmid pML-SV40-hRluc by using the EZ transfection agent (Life-iLab, Shanghai, China) for 15 h. Next, the transfected cells were concurrently induced to re-enter the cell cycle and treated with GI_90_ of safranal for a further 24 h. Thereafter, the cell lysates were collected for determination of firefly and renilla luciferase activities. Renilla luciferase served to normalize the values of the experimental reporter gene and acted as an internal control for transfection efficiency.

### Implantation of Tumor Xenografts in Nude Mice

The *in vivo* experiments were approved by the Shanghai University of Traditional Chinese Medicine and animal care was in accordance with the institutional guidelines. Five-week-old male BALB/c nude mice were sourced from the Experimental Animal Center of the Chinese Academy of Sciences (Shanghai, China) and housed in a pathogen-free environment. All mice were subcutaneously injected with 3 × 10^6^ quiescent PC-3 cells and then randomly distributed into two groups of six mice for the oral administration of the vehicle control and safranal (100 mg/kg, ig), respectively. Safranal was diluted with normal corn oil and used to pre-treat the mice a day prior to the implantation and for 46 days thereafter. The tumor size and body weight were recorded on alternate days. The mice were then sacrificed to retrieve the tumors, weighed, and photographed.

### Immunohistochemistry

The tumor tissues were fixed in 10% neutral-buffered paraformaldehyde, followed by immersion in liquid paraffin, and sectioned (5-μm thickness). Then, the samples were stained with hematoxylin and eosin and with antibodies against Ki-67 (Abcam, ab16667), NF-κB p65 (Santa Cruz, sc514451), p-IκBα (Santa Cruz, sc8404), p21 (Proteintech, #10355-1-AP), CDK4 (Epitomics, #3830-1), CDK6 (Proteintech, #14052-1-AP), CDK2 (Abcam ab32147), p-Rb (Ser807, Abcam, ab184796), E2F1 (St John’s Laboratory, STJ92807), Skp2 (Santa Cruz, sc7164), c-MYC (Abcam, ab32072), and p27 (sc528, Santa Cruz). Finally, the sections were mounted with DPX Mountant (Sigma, 317616) for histological analysis. Staining scores were noted by the intensity and percentage of positively stained cells. The percentage of positive tumor cells was divided into four grades: 0 (<5% positive), 1 (<25% positive), 2 (25–50% positive), 3 for (51–75% positive), and 4 (>75% positive). The intensity of immunostaining was scored as follows: 0 (no staining), 1 (weak staining), 2 (intermediate staining), or 3 (strong staining). Ten random fields were selected and viewed at ×400 in each section to obtain an average score (Li et al., 2020).

### Statistical Analysis

All data are presented as mean ± SD values from three independent assays. Statistical analyses were performed with SPSS 21.0 using one-way ANOVA or Student’s *t*-test. A probability value of *P* < 0.05 was considered to be statistically significant. Statistical significance was indicated as ^∗^*P* < 0.05, ^∗∗^*P* < 0.01, and ^∗∗∗^*P* < 0.001.

## Results

### Safranal Inhibits the Re-proliferation of Quiescent Pca Cells

**FIGURE 1:**
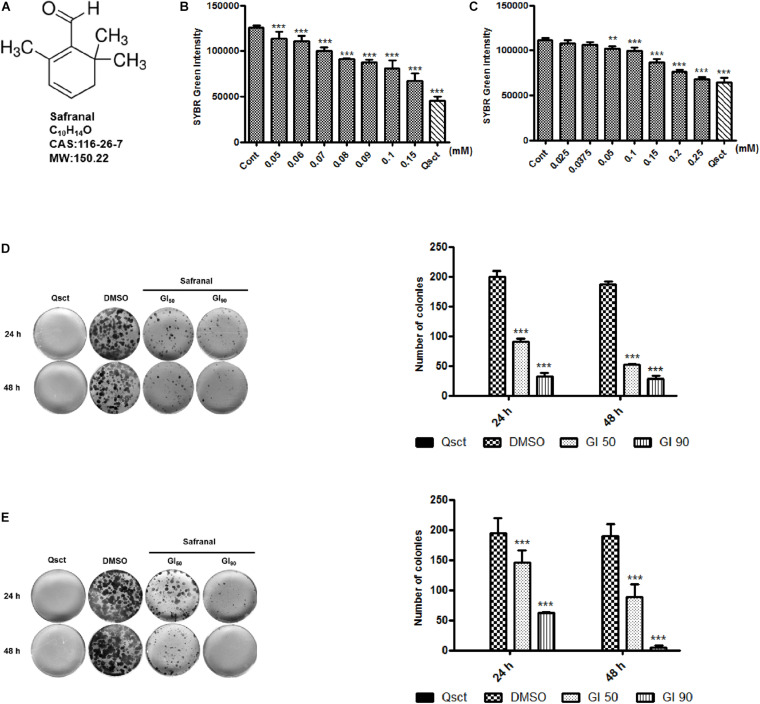
Safranal inhibits DNA synthesis and re-proliferation following induction of cell cycle re-entry. Chemical structure of safranal **(A)**. Quiescent LNCaP **(B)** and PC-3 **(C)** cells were induced to re-enter the cell cycle, treated with the indicated concentrations of safranal for 72 h, and subjected to the SYBR Green assay to assess their DNA content. Quiescent LNCaP **(D)** and PC-3 cells **(E)** were stimulated to re-enter the cell cycle in the presence or absence of safranal (GI_50_ or GI_90_) for 24 and 48 h, respectively. The cells were subsequently cultured in fresh full medium for 2 weeks, followed by ethanol fixation, crystal violet staining, and imaging. Representative images and quantification data of LNCaP and PC-3 cell colonies are presented. All data are shown as mean ± SD of triplicate experiments. **P < 0.01, ***P < 0.001 vs. vehicle control. Cont, control cells (non-quiescent cells); Qsct, quiescent cells (7-day serum withdrawal for LNCaP or 3-day contact inhibition for PC-3).

To examine the inhibitory effect of safranal (Figure 1A) on cell cycle re-entry, quiescent LNCaP cells were re-activated by serum replenishment, while quiescent PC-3 cells were reseeded at low confluency, in addition to the indicated concentrations of safranal. SYBR Green, a double-stranded DNA fluorescent dye, was applied to assess the re-synthesis of DNA content with or without safranal treatment. The DNA contents of the LNCaP control group (Figure 1B) and that of the PC-3 control cells (Figure 1C) were notably increased 72 h after re-activation from quiescence. Safranal decreased the DNA re-synthesis of quiescent LNCaP and PC-3 cells in a dose-dependent manner compared with the control group at 72 h, indicative of the inhibition of QCC re-proliferation. The concentrations of safranal-mediated growth inhibition (GI) at 50% (GI_50_) and 90% (GI_90_) in LNCaP and PC-3 cells were established based on the SYBR Green assay results (Table 1). Additionally, we monitored the cytotoxicity of safranal on non-malignant prostate stromal cell line WPMY-1, human normal liver cell line HL-7702, and proliferative LNCaP and PC-3 cells (IC_50_ values listed in the Supplementary Table 1). Safranal was less cytotoxic to the two normal human cell lines and exhibited better inhibitory effect on quiescent Pca re-proliferation compared to the proliferative LNCaP and PC-3 cells. Then, we determined the prolonged effectiveness of safranal on inhibiting cell cycle re-entry using the colony formation assay. Quiescent LNCaP (Figure 1D) and PC-3 cells (Figure 1E) were released from quiescence and treated with GI_50_ or GI_90_ of safranal for 24 and 48 h and then maintained in a fresh medium without safranal for an additional 2 weeks. Safranal exerted a long-term effect on Pca re-proliferation and significantly decreased the number and size of colonies in a dose- and time-dependent manner. Overall, these data suggest that safranal exerts an inhibitory effect on quiescent Pca re-proliferation.

### Safranal Blocks Quiescent Pca Cells From Re-entering Cell Cycle by Downregulating G_0_/G_1_-Related Proteins

**FIGURE 2:**
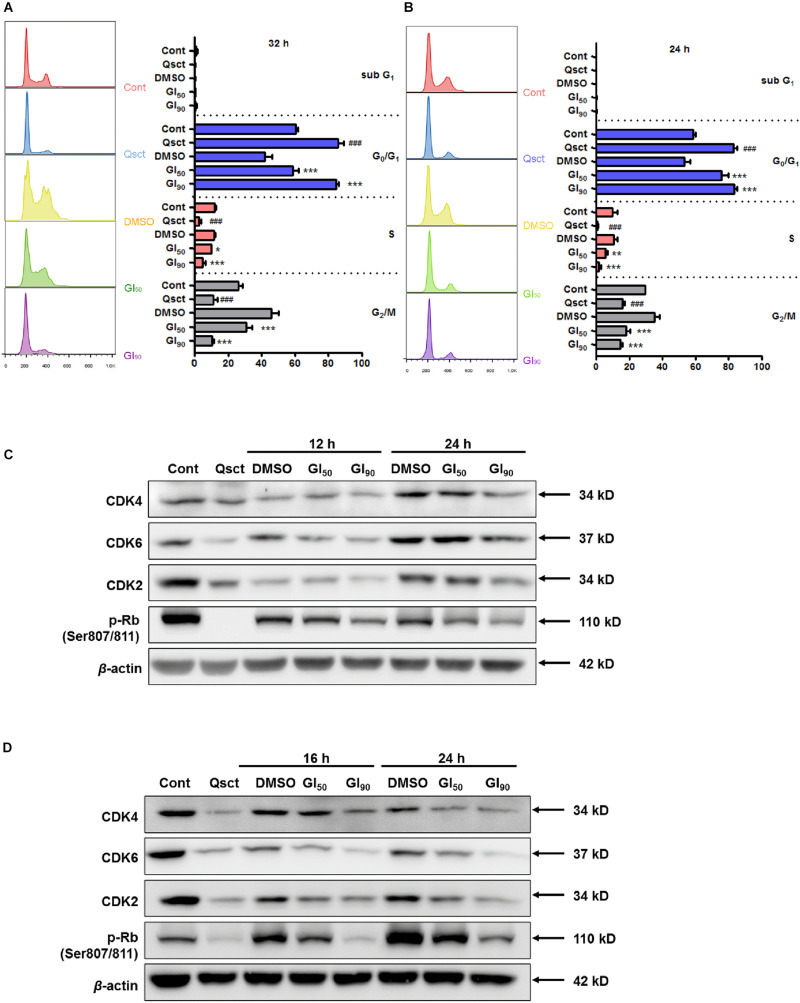
Safranal prevents quiescent Pca cell re-entry by downregulating G_0_/G_1_-related proteins. Quiescent LNCaP **(A)** and PC-3 **(B)** cells were triggered to re-enter the cell cycle in the presence of dimethyl sulfoxide (DMSO) or safranal (GI_50_ or GI_90_). The cells were harvested after 32 and 24 h, respectively, followed by PI staining, and subjected to flow cytometry. Immunoblotting detected the protein levels of CDK2, CDK4, CDK6, and p-Rb (Ser807/811) in treated LNCaP **(C)** and treated PC-3 cells **(D)** during cell cycle re-entry at the indicated intervals. β-actin was used as a loading control. Data are shown as mean ± SD of triplicate experiments. ^###^*P* < 0.001 vs. non-quiescent control or *P < 0.05, **P < 0.01, ***P < 0.001 vs. DMSO vehicle control.

We investigated the role of safranal on cell cycle progression by using PI staining flow cytometric analysis, and we monitored the cell cycle distribution after release from quiescence in the presence or absence of safranal for 32 and 24 h in LNCaP and PC-3 cells, respectively. Experimental quiescence was achieved by a 7-day serum withdrawal for LNCaP cells and 3-day contact inhibition for PC-3 cells, which resulted in quiescence with 85.9% of LNCaP (Figure 2A) and 83.0% of PC-3 cells (Figure 2B) in the G_0_/G_1_ phase. Following the re-entry of Pca cells, safranal at GI_90_ maintained cells in the G_0_/G_1_ phase as 84.5% of LNCaP and 83.0% of PC-3 cells approached the level of quiescent cells. In contrast, the control LNCaP and PC-3 cells readily re-entered the cell cycle after leaving quiescence. Our findings imply that safranal significantly retards the cell cycle progression of quiescent Pca cells compared with the DMSO control in a dose- and time-dependent manner (Figures 2A,B).

Activation of G_0_/G_1_ phase-related cyclins and CDK complexes and phosphorylation of retinoblastoma protein (Rb) cause E2F release and promote cell cycle progression (So and Cheung, 2018). According to the preliminary study, G_0_/G_1_ phase-related proteins were altered prior to cell cycle distribution, as monitored by flow cytometry. The protein expression levels of CDK2, CDK4, CDK6, and phospho-Rb (Ser807/811) declined in quiescent Pca cells (Figures 2C,D), and almost all of them began to recover at 12 and 16 h after quiescent LNCaP and PC-3 cell re-entry into the cell cycle, respectively. Additionally, safranal significantly suppressed the recovery of these proteins upon resumption of the cell cycle in these cell lines. Overall, these results suggest that safranal blocks quiescent Pca cells from re-entering the cell cycle by downregulating the G_0_/G_1_-related proteins.

### Safranal Downregulates c-MYC/Skp2/p27 During Cell Cycle Re-entry

**FIGURE 3:**
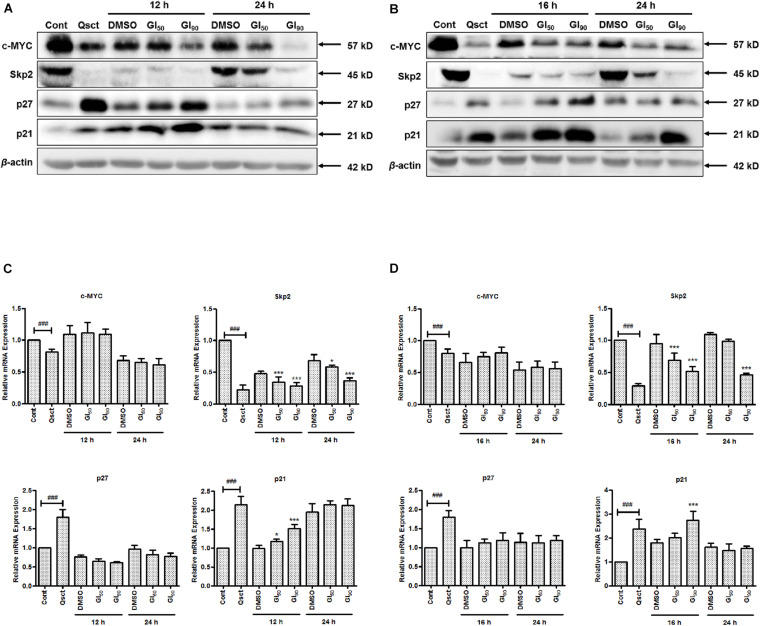
Safranal downregulates the post-transcription of c-MYC and the transcription of *Skp2* during cell cycle re-entry in Pca cells. Quiescent LNCaP **(A,C)** and PC-3 **(B,D)** cells were stimulated to re-enter the cell cycle in the presence or absence of safranal (GI_50_ or GI_90_) at the indicated intervals. The protein and mRNA levels of c-MYC, Skp2, p21, and p27 were then analyzed using immunoblotting **(A,B)** and RT-PCR **(C,D)** at each interval. β-actin served as a loading control. Cont, control cells in a proliferative state. Results are expressed as mean ± SD of triplicate assays. ^###^*P* < 0.001 vs. non-quiescent control or *P < 0.05, ***P < 0.001 vs. dimethyl sulfoxide vehicle control.

To determine the underlying mechanism of inhibition on quiescent Pca cell cycle re-entry, we examined the protein and mRNA expression levels of c-MYC, Skp2, and CDK inhibitors, namely, p27 and p21, in the presence or absence of safranal. Compared with non-quiescent cells, the protein and mRNA expression levels of c-MYC and Skp2 were significantly decreased in quiescent Pca cells and gradually recovered after release from quiescence. Safranal considerably decreased the protein expression levels of c-MYC and Skp2 in LNCaP (Figure 3A) and PC-3 (Figure 3B) cells. Moreover, the protein levels of p21 and p27, as downstream targets of c-MYC, were dramatically accumulated in the quiescent state and gradually decreased after the cells were released from quiescence. However, safranal significantly promoted the accumulation of p21 in LNCaP and p27 in PC-3 cells after 12 and 16 h, respectively, but not at a later time point (Figures 3A,B). At the mRNA level, safranal notably increased p21 levels after 12 and 16 h in LNCaP and PC-3 cells, respectively, without the concomitant regulation of c-MYC and p27 (Figures 3C,D). Skp2, a member of F-box proteins, was involved in coordinating the G_1_/S transition and cancer progression. Therefore, we evaluated the mRNA and protein changes of Skp2 and observed that safranal significantly downregulated Skp2 at the transcriptional and protein levels (Figures 3A–D), suggesting that it deregulates Skp2 transcription. These data show that safranal downregulates post-transcriptional c-MYC and the transcription of Skp2, which collectively impairs the degradation of the CDK inhibitors p27 and p21. Hence, further investigation into the underlying mechanism of safranal-induced deregulation of *Skp2* was conducted.

### Safranal Reduces AKT Phosphorylation and Suppresses E2F1 Transcriptional Activity

**FIGURE 4:**
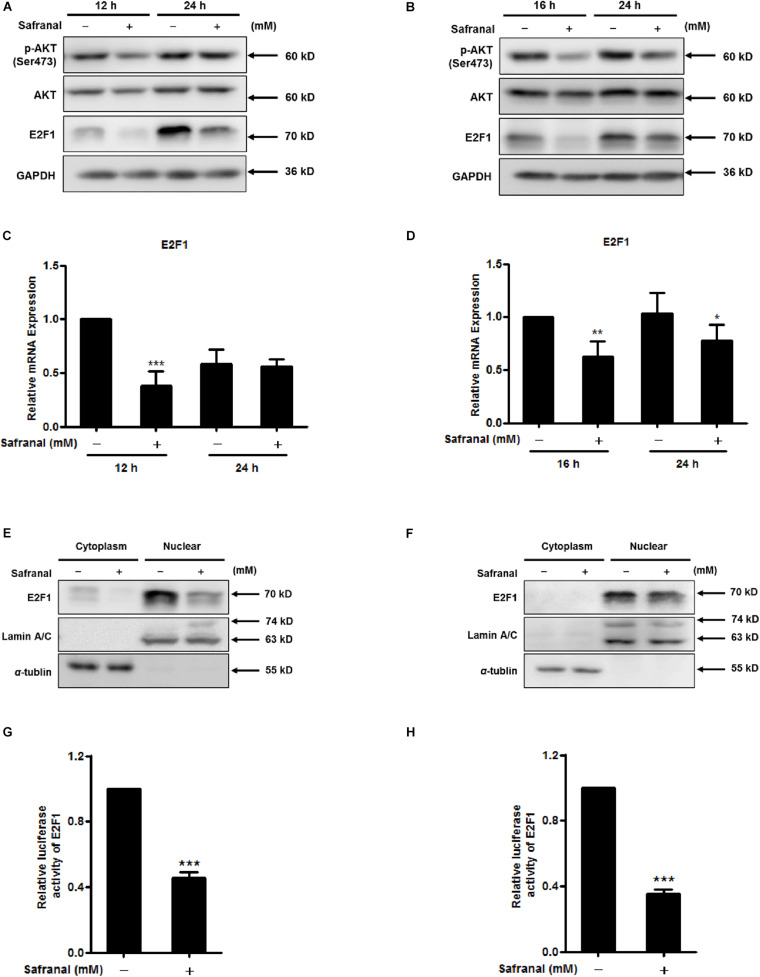
The inhibitory effects of safranal on AKT phosphorylation and transcriptional activity of E2F1 during quiescent Pca cell re-entry. Quiescent LNCaP **(A)** and PC-3 **(B)** cells were initially stimulated to re-enter the cell cycle, and the effects of safranal (GI_90_) on protein expression levels of p-AKT (Ser473), AKT, and E2F1 at specific intervals were determined by immunoblotting. GAPDH served as a loading control. The mRNA expression levels of E2F1 in LNCaP **(C)** and PC-3 **(D)** cells during cell cycle re-entry in the presence or absence of safranal (GI_90_) were examined by RT-qPCR. E2F1 levels in cell nuclear and cytoplasm extracts of LNCaP **(E)** and PC-3 cells **(F)** were analyzed using immunoblotting after treatment with safranal for 12 and 3 h, respectively, following release from the quiescent state. α-Tubulin and lamin A/C served as loading and purity controls for the cytoplasm and nuclear fractions, respectively. Following a 6-day serum withdrawal for LNCaP and 2-day contact inhibition for PC-3 cells, quiescent LNCaP **(G)** and PC-3 **(H)** were transfected with pGM-E2F-Luc and renilla luciferase reporter plasmid pML-SV40-hRluc by using the EZ transfection agent for 15 h. The transfected quiescent cells were then induced to re-enter the cell cycle in the presence or absence of safranal (GI_90_) for 24 h. Cell lysates were collected to assess firefly and renilla luciferase activities using the dual luciferase reporter assay kit. Renilla luciferase served to normalize the values of the experimental reporter gene and acted as an internal control for transfection efficiency. Data are expressed as mean ± SD. *P < 0.05, **P < 0.01, ***P < 0.001 vs. dimethyl sulfoxide vehicle control.

PI3K/AKT signaling positively regulates Skp2 transcription by coupling with E2F1, a critical Skp2 transcription activator (Reichert et al., 2007; Obinata et al., 2017). We evaluated phospho-AKT and E2F1 protein levels during re-entry of quiescent LNCaP (Figure 4A) and PC-3 cells (Figure 4B). Phosphorylation at Ser473 activated AKT (Freudlsperger et al., 2015), safranal inhibited E2F1 and p-AKT (Ser473), and the expression of total AKT remained unchanged. Safranal significantly lowered the mRNA levels of E2F1 at 12 h after the LNCaP cells were released from quiescence, although this was less apparent after 24 h (Figure 4C). In PC-3 cells, E2F1 mRNA levels decreased after 16 and 24 h of cell cycle re-entry (Figure 4D).

To examine the mediatory effect of safranal on nuclear translocation of E2F1 during cell cycle re-entry, nuclear and cytosolic fractions were separated. Safranal decreased the protein expression level of E2F1, which was predominantly distributed in the nucleus of LNCaP and PC-3 cells after release from quiescence (Figures 4E,F). To further investigate the inhibitory effect of safranal on the transcriptional activity of E2F1 during quiescent Pca cell re-entry, dual luciferase reporter assay was performed. Safranal significantly decreased E2F transcriptional activity in LNCaP (Figure 4G) and PC-3 cells (Figure 4H) during QCCs’ re-entry. Overall, these results suggest that safranal reduces AKT phosphorylation and suppresses E2F transcriptional activity.

### Safranal Deregulates the Canonical and Non-canonical NF-κB Signaling Pathways

**FIGURE 5:**
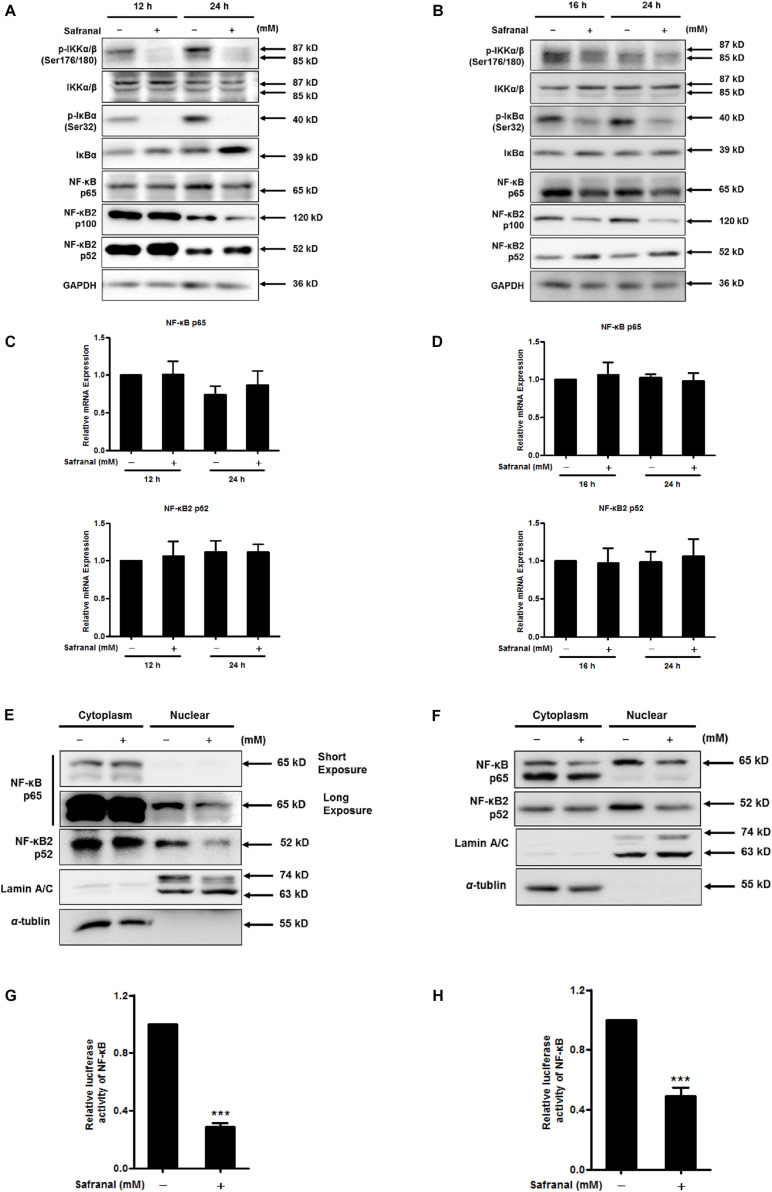
The inhibitory effect of safranal on the activation of the canonical and non-canonical NF-κB pathways during quiescent Pca cell re-entry. Following the induction of quiescent LNCaP **(A)** and PC-3 **(B)** cell re-entry into the cell cycle, the effects of safranal (GI_90_) on the protein expression levels of p-IKKα/β (Ser176/180), IKKα/β, p-IκBα (Ser32), IκBα, NF-κB p65, and p100/p52 at the indicated intervals were determined by immunoblotting. GAPDH served as a loading control. The mRNA expression levels of p65 and p52 in LNCaP **(C)** and PC-3 **(D)** cells during cell cycle re-entry in the presence or absence of safranal (GI_90_) were assessed by RT-qPCR. NF-κB p65 and p52 levels in cell nuclear and cytoplasm extracts of LNCaP **(E)** and PC-3 cells **(F)** were analyzed by immunoblotting after treatment with safranal for 24 and 1 h, respectively, following release from the quiescent state. α-Tubulin and lamin A/C were used as loading and purity controls for the cytoplasm and nuclear fractions, respectively. Following a 6-day serum withdrawal for LNCaP **(G)** and a 2-day contact inhibition for PC-3 cells **(H)**, the cells were transfected with pML-NFκB-Fluc2-Neo enhanced and renilla luciferase reporter plasmid pML-SV40-hRluc by using the EZ transfection agent for 15 h. The transfected quiescent cells were then induced to re-enter the cell cycle in the presence or absence of safranal (GI_90_) for 24 h. Cell lysates were collected to determine firefly and renilla luciferase activities using the dual luciferase reporter assay kit. Renilla luciferase served to normalize the values of the experimental reporter gene and as an internal control for transfection efficiency. Data are expressed as mean ± SD. ***P < 0.001 vs. dimethyl sulfoxide vehicle control.

NF-κB is another crucial transcriptional activator of Skp2, as blocking IKK/NF-κB turns off *Skp2* gene expression (Schneider et al., 2006). IκBs is a switch protein of NF-κB that regulates the canonical NF-κB pathway of NF-κB activation. Once IκBα is phosphorylated by IKK, various NF-κB complexes are translocated to the nucleus, predominantly the p50/RelA dimer. In particular, a non-canonical NF-κB pathway relies on the inducible processing of p100 rather than the degradation of IκBα, thus activating the RelB/p52 NF-κB complex and the downstream target *Skp2* (Richmond, 2002). To determine the repressive effect of safranal on NF-κB pathway in the downregulation of Skp2 transcription during the re-entry of quiescent Pca cells into the cell cycle, the transcriptional activity of NF-κB and its corresponding upstream regulators were examined. Safranal notably hindered the phosphorylation of IKKα/β (Ser176/180) without affecting the total protein expression in LNCaP (Figure 5A) and PC-3 cells (Figure 5B). Furthermore, safranal upregulated the protein expression of IκB and substantially inhibited the phosphorylation of IκB (Ser32), and it ultimately downregulated the protein expression of NF-κB p65. Additionally, we examined the expression of p100 NF-κB2 and its processed product p52. Notably, safranal-induced p52 expression was associated with a decrease in the level of the precursor p100, particularly after 24 h in both cell lines. Additionally, we verified that the mRNA level of p65 and p52 remained unchanged post-safranal exposure in LNCaP (Figure 5C) and PC-3 cells (Figure 5D).

Nuclear translocation of NF-κB subunits is the hallmark of NF-κB activity. An evaluation of cell nuclear and cytoplasmic extracts indicated that safranal reduced the protein expression of nuclear NF-κB p65 and p52 during the re-entry of quiescent LNCaP (Figure 5E) and PC-3 cells (Figure 5F). This suggested that safranal deregulated the canonical and non-canonical NF-κB signaling pathways. To further investigate the effect of safranal on NF-κB transcriptional activity during quiescent Pca cell re-entry, dual luciferase reporter assay was performed. Safranal suppressed the NF-κB transcriptional activity after quiescent LNCaP (Figure 5G) and PC-3 cells (Figure 5H) re-entered the cell cycle. Overall, these data support that safranal inhibits the canonical and non-canonical NF-κB signaling pathways during cell cycle re-entry of Pca cells.

### The Potential Effect of Safranal on Suppressing Pca Recurrence *in vivo*

**FIGURE 6:**
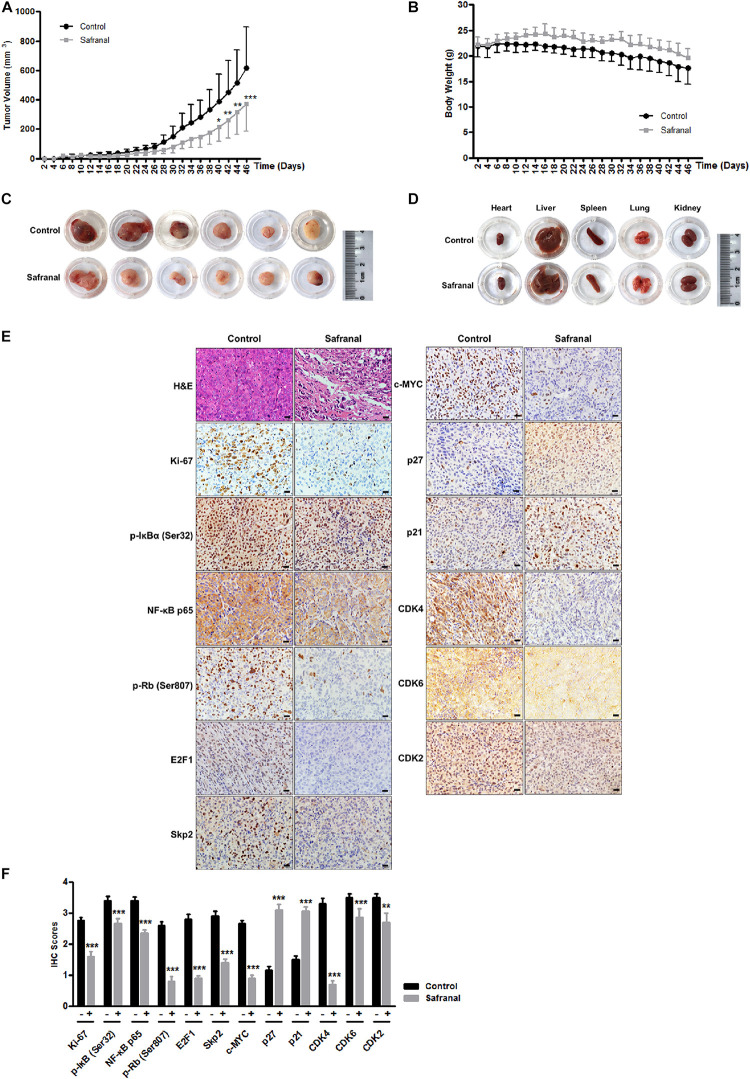
Safranal suppresses the tumor growth of quiescent PC-3 cells *in vivo*. Quiescent PC-3 cells were xenografted subcutaneously into the left flank of male nude mice. Then, the mice were orally administrated with either the vehicle control or 100 mg/kg of safranal, commencing a day prior to the xenograft procedure. Tumor volume **(A)** and mice body weight **(B)** were measured on alternate days. After a 46-day treatment, the mice were sacrificed, and the tumor **(C)** and major organs **(D)** were resected and photographed (scale bar: 20 μm). Paraffin-embedded tumor tissues were stained with hematoxylin and eosin and with antibodies against Ki-67, Skp2, E2F1, NF-κB p65, p-IκBα (Ser32), c-MYC, p-Rb (Ser807), CDK4, CDK6, CDK2, E2F1, Skp2, p21, and p27; representative images (**E**, ×400) and quantification data **(F)** are presented. All data are shown as mean ± SD. *P < 0.05, **P < 0.01, ***P < 0.001 vs. control.

The re-awakening of QCCs is considered as the main reason of cancer recurrence and progression. Blocking the transition of cancer cells from quiescence to proliferation is critical to prevent cancer recurrence. To further corroborate the potential effects of safranal on suppressing the re-proliferation of quiescent Pca *in vivo*, we established the xenograft model of quiescent PC-3 cells to mimic the progression of cancer recurrence. All mice were subcutaneously injected with 3 × 10^6^ quiescent PC-3 cells and then randomly distributed into two equal groups of six mice and orally administered with the vehicle control and safranal (100 mg/kg, ig), respectively. Safranal treatment commenced a day prior to tumor inoculation. Daily treatment of safranal significantly suppressed the tumor growth (Figure 6A) despite the unapparent change in the mice body weight (Figure 6B) or pathology of major organs (Figure 6D) compared with the control group. After 46 days of treatment, the tumors in the safranal-treated group (average: 372 mm^3^) were markedly smaller than those in the control group (average: 618 mm^3^), with an average reduction of 40% (Figures 6A,C). Moreover, safranal delayed prostate tumor recurrence as the time required for tumor to attain 150 mm^3^ in the safranal group (38 days) was considerably longer than in the control group (30 days), with an average increase of 26.7%. An examination of resected tumors revealed that the safranal-treated mice had sparse cell density and less Ki-67 positivity (Figures 6E,F). We also confirmed that safranal treatment downregulated the protein expression levels of NF-κB p65, p-IκBα (Ser32), E2F1, Skp2, c-MYC, p-Rb (Ser807), CDK4, CDK6, and CDK2 and elevated p21 and p27 expression in safranal-treated tumor tissues compared with the vehicle control (Figures 6E,F), which is in agreement with the *in vitro* findings. These data suggest that safranal potentially suppresses Pca recurrence *in vivo*, reduces Skp2, E2F1, NF-κB p65, p-IκBα (Ser32), c-MYC, p-Rb (Ser807), CDK4, CDK6, and CDK2, and elevates p21 and p27 in safranal-treated tumor tissues.

## Discussion

It is well-known that re-entry of QCCs into the cell cycle results in cancer recurrence and metastasis (Chen et al., 2012; Li et al., 2015; Tejero et al., 2019). As F-box protein Skp2 is one of the key regulators in cell cycle re-entry (Bashir et al., 2004; Chan et al., 2010), its mRNA and protein expression is at its lowest in the quiescent state and increases as the cell cycle transits to the G_1_/S phase (Wirbelauer et al., 2000; Bashir et al., 2004), which was also verified in our present study. Safranal downregulates mRNA and protein levels of Skp2 during cell cycle re-entry, which plausibly reduces the risk of Pca recurrence. Further mechanism studies showed that the transcriptional activities of two major transcriptional activators of Skp2, E2F1, and NF-κB were possibly decreased through AKT phosphorylation and the inhibition of the canonical and non-canonical NF-κB signaling pathways. Our *in vivo* experiments confirm that safranal inhibits the tumorigenicity of quiescent PC-3 cells and represses tumor growth, indicating its potential in suppressing Pca recurrence.

AKT and E2F1 signaling plays a critical role in the proliferation and survival of Pca cells (Suh and Rabson, 2004). The findings of this study indicate that safranal downregulates the expression of p-AKT (ser473), an active site of AKT. Additionally, safranal decreases the mRNA and protein levels of E2F1 in the nucleus and the transcriptional activity of E2F. E2F1 acts as a transcription factor for *Skp2* and other genes important for G_1_/S shift in cell cycle progression (Reichert et al., 2007). When quiescent cells are stimulated to re-enter the cell cycle, there is a surge in the protein levels and activity of E2F1, reaching its peak in late G_1_ phase to modulate transition to the S phase (Dubrez, 2017). Therefore, inhibition of E2F1 suppresses the re-activation of QCCs and the further advancement of cells to the S phase, exerting multiple inhibitory effects on tumor recurrence and progression. NF-κB, a major nuclear transcription factor involved in the progression of Pca (Sweeney et al., 2004; Shukla et al., 2005) and its inhibition in the bone marrow microenvironment, induces quiescence in breast cancer cells (Ramkissoon et al., 2007). Impairment of NF-κB suppresses the quiescent cancer cells from re-entering the cell cycle, thus preventing its progression to cancer recurrence and metastasis. The NF-κB canonical pathway is triggered by the degradation of IκBα *via* phosphorylation, releasing sequestered NF-κB complexes (predominantly the p65/p50 dimer) for nuclear translocation (Jeon et al., 2017), followed by the activation of the transcription of Skp2 (Schneider et al., 2006). Additionally, the non-canonical NF-κB pathway largely relies on p100 processing following the nuclear translocation of the p52-RelB dimer. Our data suggest that safranal blocks the canonical and non-canonical NF-κB pathways by deregulating IκB phosphorylation and p100 processing, inhibiting the nuclear translocation of p65 and p52 and thus blocking the transcription of Skp2. Further gain- or loss-of-function studies are required to determine the effect of safranal on PI3K/AKT signaling and NF-κB pathway in regulating cancer recurrence in relation to *Skp2* suppression.

## Conclusion

In conclusion, we have elucidated that safranal suppresses the re-proliferation of quiescent Pca cells *in vitro* and exerts a long-term inhibitory effect on clonogenic formation. Mechanism studies showed that safranal inhibits *Skp2* transcription, possibly by suppressing the transcriptional activity of E2F1, and NF-κB subunits. Our *in vivo* study demonstrated that safranal delays the re-growth of quiescent PCa and inhibits tumor progression *via* the downregulation of Skp2, E2F1, NF-κB p65, p-IκBα (Ser32), c-MYC, p-Rb (Ser807), CDK4, CDK6, and CDK2 expression and elevation of p21 and p27 levels in tumor tissues, concordant with our *in vitro* findings. Therefore, our study highlights safranal as a potential therapeutic agent for Pca recurrence and provides evidence that pharmacological inactivation of the NF-κB/E2F1–Skp2 axis is a potential therapeutic target against cancer recurrence and progression.

## Data Availability Statement

All datasets generated for this study are included in the article/Supplementary Material, further inquiries can be directed to the corresponding authors.

## Ethics Statement

The animal study was reviewed and approved by the Shanghai University of Traditional Chinese Medicine.

## Author Contribions

ZX and HX designed and conceived the study. XJ and YaL performed the experiments and analyzed the data with the assistance of JF. RW and YuL provided technical support in the experiments. YaL and ZX drafted the manuscript. WN and HL revised the manuscript. All the authors approved the final version and agreed for its publication.

## Conflict of Interest

The authors declare that the research was conducted in the absence of any commercial or financial relationships that could be construed as a potential conflict of interest. The handling editor TL declared a past co-authorship with several of the authors ZX and HX.

## Abbreviations

CDK: cyclin-dependent kinase
CI: confidence interval
GI: growth inhibition
PBS: phosphate-buffered saline
Pca: prostate cancer
PI: propidium iodide
QCCs: quiescent cancer cells
RT-qPCR: real-time reverse transcription PCR
Rb: retinoblastoma protein
Skp2: S-phase kinase-associated protein 2.
