# Supplementary material for: Safrana l Prevents Prostate Cancer Recurrence by Blocking the Re-activation of Quiescent Cancer Cells via Downregulation of S-Phase Kinase-Associated Protein 2
Source: Front Cell Dev Biol. 2020 Dec 16;8:598620. doi: 10.3389/fcell.2020.598620 (PMC7772204; doi:10.3389/fcell.2020.598620)
Supplement: Supplementary file 1 [file Data_Sheet_1.DOCX]

Supplementary Material

# Supplementary materials and methods

## Cell lines

Non-malignant prostate stromal cell line WPMY-1, and human normal liver cell line HL-7702, were acquired from the American Type Culture Collection (Manassas, VA, USA).

## Cell viability assay

One day after seeding in 96-well plates, cells were exposed to different concentrations of Safranal for 48 h. Cell viability was measured using the Cell Counting Kit (CCK8) (Yeasen, China), and the absorbance was measured at 470 nm using a microplate reader (FLUOstar Omega, BMG Labtech, Germany). The results represent the mean ± SD of at least three independent experiments.

# Supplementary data

The raw data of Figure 2A was provided as supplementary data (Excel file).

# Supplementary Figures and Tables

## Supplementary Table

**Supplementary Table 1.** IC_50_ values of Safranal in non-malignant prostate stromal cell line WPMY-1, human normal liver cell line HL-7702, and proliferative LNCaP and PC-3 cells.

| **Cell line** | **IC_50_ (mM)** |
| --- | --- |
| WPMY-1 | 0.507 ± 0.082 |
| HL-7702 | 1.657 ± 0.147 |
| LNCaP | 0.234 ± 0.023 |
| PC-3 | 0.512 ± 0.038 |

IC_50_ values of Safranal were determined with CCK8 assay in WPMY-1, HL-7702, LNCaP, and PC-3 cells at different concentrations for 48 h. The results represent the mean ± SD of at least three independent experiments.

## Supplementary Figures


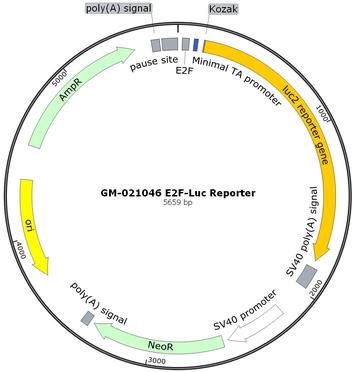
**A**


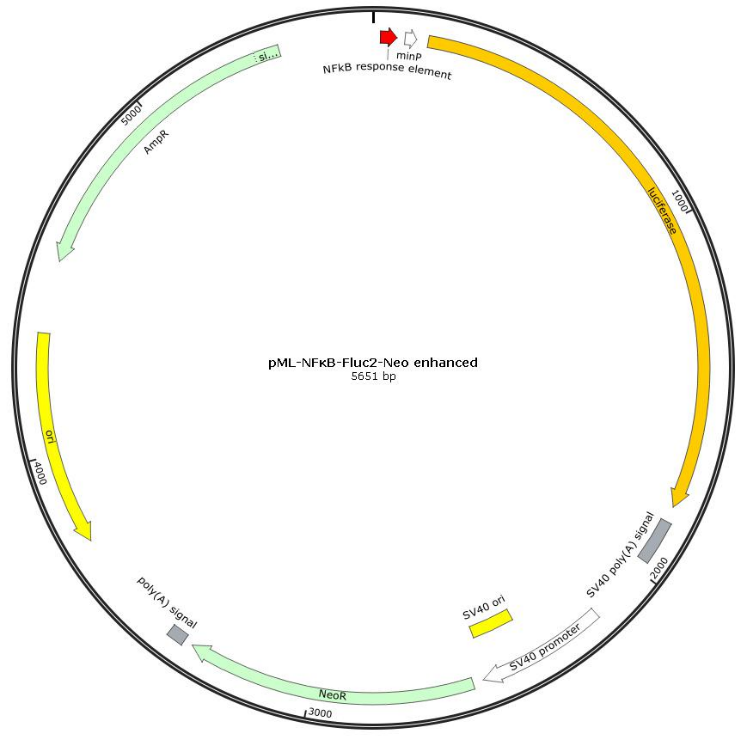
**B**


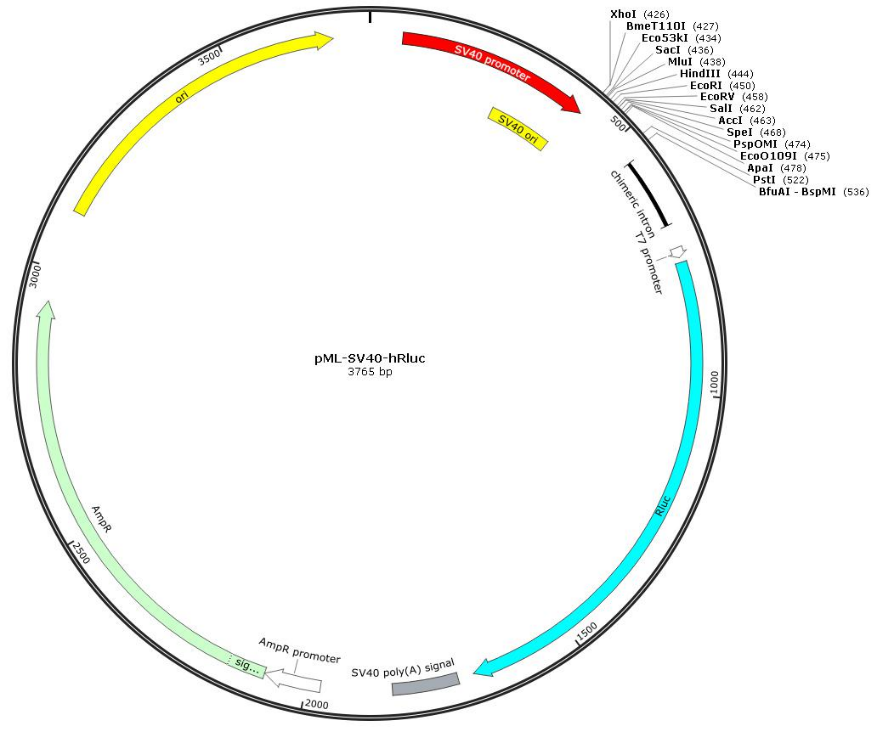
**C**

**Supplementary Figure 1.** The schematic diagram of E2F-Luc (A), pML-NFκB-Fluc2-Neo enhanced (B) and pML-SV40-hRluc (C) reporter constructs.
